# Common and distinct neural effects of risperidone and olanzapine during procedural learning in schizophrenia: a randomised longitudinal fMRI study

**DOI:** 10.1007/s00213-015-3959-1

**Published:** 2015-05-19

**Authors:** Veena Kumari, Ulrich Ettinger, Seoung Eun Lee, Christine Deuschl, Anantha P. Anilkumar, Anne Schmechtig, Philip J. Corr, Dominic H. ffytche, Steven C. R. Williams

**Affiliations:** Department of Psychology, P078, Institute of Psychiatry, Psychology and Neuroscience, King’s College London, De Crespigny Park, London, SE5 8AF UK; National Institute for Health Research Biomedical Research Centre for Mental Health, Institute of Psychiatry, London, UK; Department of Psychology, University of Bonn, Bonn, Germany; South London and Maudsley NHS Foundation Trust, London, UK; Department of Neuroimaging, Institute of Psychiatry, Psychology and Neuroscience, King’s College London, London, UK; Department of Psychology, City University, London, UK; Department of Old Age Psychiatry, Institute of Psychiatry, Psychology and Neuroscience, King’s College London, London, UK

**Keywords:** Sequence learning, Striatum, Anterior cingulate, Typical antipsychotics, Atypical antipsychotics

## Abstract

**Rationale:**

Most cognitive domains show only minimal improvement following typical or atypical antipsychotic treatments in schizophrenia, and some may even worsen. One domain that may worsen is procedural learning, an implicit memory function relying mainly on the integrity of the fronto-striatal system.

**Objectives:**

We investigated whether switching to atypical antipsychotics would improve procedural learning and task-related neural activation in patients on typical antipsychotics. Furthermore, we explored the differential effects of the atypical antipsychotics risperidone and olanzapine.

**Methods:**

Thirty schizophrenia patients underwent functional magnetic resonance imaging during a 5-min procedural (sequence) learning task on two occasions: at baseline and 7–8 weeks later. Of 30 patients, 10 remained on typical antipsychotics, and 20 were switched randomly in equal numbers to receive either olanzapine (10–20 mg) or risperidone (4–8 mg) for 7–8 weeks.

**Results:**

At baseline, patients (all on typical antipsychotics) showed no procedural learning. At follow-up, patients who remained on typical antipsychotics continued to show a lack of procedural learning, whereas those switched to atypical antipsychotics displayed significant procedural learning (*p* = 0.001) and increased activation in the superior-middle frontal gyrus, anterior cingulate and striatum (cluster-corrected *p* < 0.05). These neural effects were present as a linear increase over five successive 30-s blocks of sequenced trials. A switch to either risperidone or olanzapine resulted in comparable performance but with both overlapping and distinct task-related activations.

**Conclusions:**

Atypical antipsychotics restore procedural learning deficits and associated neural activity in schizophrenia. Furthermore, different atypical antipsychotics produce idiosyncratic task-related neural activations, and this specificity may contribute to their differential long-term clinical profiles.

## Introduction

Schizophrenia is a complex neurobiological disorder thought to arise from a dysregulation of multiple neurotransmitters including the dopaminergic, glutamatergic and serotonergic systems (Coyle 2006; Davis et al. 1991; Geyer and Vollenweider 2008; Howes and Kapur 2009; Javitt and Zukin 1991; van Rossum 1966). In addition to the core symptoms that form the diagnostic criteria, cognitive impairment has been increasingly described as a prominent feature of schizophrenia that should be a target for treatment (Carter and Barch 2007), given its relationship with poor functional outcome and no established treatment so far (Harvey and Bowie 2012).

Existing pharmacological treatments include typical antipsychotics and, more recently, atypical antipsychotics. Typical antipsychotics, such as fluphenazine, haloperidol and chlorpromazine, may alleviate psychotic symptoms through the blockade of dopamine D_2_ receptors in the striatum (Abi-Dargham 2004; Laruelle and Abi-Dargham 1999; Peroutka and Synder 1980). Commonly prescribed atypical antipsychotics such as risperidone and olanzapine act on multiple neurotransmitter systems (Kapur et al. 1998; Nord and Farde 2011). Risperidone has high affinity for both serotonin 2A receptor (5-HT_2A_) and dopamine D_2_ receptors and modest affinity for histamine and alpha-2 (α_2_) adrenergic receptors (Leysen et al. 1994)*.* Olanzapine has high affinity for 5-HT_2A_ and moderate affinity for D_2_, muscarinic M_1–4_, α_1–2_ and histamine H_1_ receptors (Arnt and Skarsfeldt 1998). Furthermore, most atypical antipsychotics occupy D_2_ receptors only transiently and dissociate rapidly to allow normal dopamine neurotransmission (Seeman 2002).

Although antipsychotics were synthesised primarily to attenuate psychotic symptoms, their clinical benefits and/or side effects may extend to cognitive performance, and this needs to be examined with specific cognitive paradigms on both the behavioural and the neural level (Honey and Bullmore 2004). The use of atypical antipsychotics has been reported to alleviate cognitive impairment, at least to some degree (Woodward et al. 2005), although studies comparing the effects of typical and atypical studies have been less conclusive (Carpenter and Conley 2007; Green et al. 2002; Keefe et al. 2007b; Meltzer 2004). While it is desirable that patients demonstrate generalised cognitive improvement, there is also a need to find out whether any of the specific cognitive deficits result from, or become exaggerated by, antipsychotic treatments (Heinrichs 2007; Hill et al. 2010).

Due to the multi-factorial nature of most traditional neuropsychological tests, they offer limited sensitivity to specific cognitive changes induced by antipsychotics (Carter and Barch 2007). Procedural learning (PL), which refers to the gradual acquisition of skills through repeated exposure of a specific rule-governed activity (Cohen and Squire 1980), may provide a useful measure in this context. There is consistent evidence that PL is sensitive to dopaminergic changes, particularly in the striatum (Foerde and Shohamy 2011). The functional integrity of the striatum has been shown to be critical for PL particularly when assessed using the serial reaction time task (SRTT) or one of its variants (Doyon et al. 1998; Knopman and Nissen 1991; Nissen and Bullemer 1987; Rauch et al. 1997). SRTT is a visuospatial tracking task in which participants gradually acquire the pattern of repeating sequences amongst embedded random blocks without developing explicit awareness of their learning (Howard and Howard 1997). The learning of sequences results in faster reaction time (RT) for the sequence trials compared to the random trials, reflecting PL.

A meta-analysis revealed a moderate degree of impairment in PL on the SRTT in patients with schizophrenia relative to controls with a pooled effect size of 0.51 (Siegert et al. 2008). The studies, however, varied in medication and patient characteristics. The potential influence of antipsychotics on SRTT performance is indicated by healthy volunteer studies where otherwise normal performance is compromised by acute administration of haloperidol (Kumari et al. 1997) and chlorpromazine (Danion et al. 1992) but enhanced by the indirect dopamine agonist *d*-amphetamine (Kumari et al. 1997). Absence of PL on the SRTT has been observed in patients on typical antipsychotics (Kumari et al. 2002) but not when they are on atypical antipsychotics (Kumari et al. 2008; Stevens et al. 2002). Medication-naïve first-episode patients, and recent-onset patients with minimal prior exposure to antipsychotics, show lower learning profiles and larger inter-trial fluctuations, but no robust impairment in SRTT performance (Kumari et al. 2008; Purdon et al. 2011).

Parallel to these behavioural findings, there is evidence from a single-photon emission computed tomography (SPECT) study that PL deficits induced by haloperidol may be dopaminergically mediated in the striatum (Paquet et al. 2004). Functional magnetic resonance imaging (fMRI) studies implementing variants of the SRTT have repeatedly shown aberrant fronto-striatal activations in schizophrenia relative to healthy groups (Kumari et al. 2008; Kumari et al. 2002; Purdon et al. 2011; Reiss et al. 2006; Zedkova et al. 2006). Patients on typical antipsychotics show fronto-striatal activation deficits (Kumari et al. 2002), whereas those on ziprasidone (Kumari et al. 2008) and unmedicated first-episode patients show activation patterns broadly similar to that of healthy controls (Purdon et al. 2011). It is thus likely that the use of typical antipsychotics gives rise to commonly observed PL deficits, possibly in addition to an underlying neural abnormality affecting many cognitive functions in schizophrenia. No study has yet investigated the neural changes accompanying the effects of switching from typical to atypical antipsychotics in PL on the SRRT in schizophrenia patients. Investigating changes following a switch from typical to atypical treatment can not only clarify the impact of typical and atypical antipsychotic treatment on PL but also provide new insights into the differential neural effects of atypical antipsychotics.

The primary aim of this study was to apply fMRI in a longitudinal within-subjects design (pre- and post-) to detect changes in the blood-oxygen-level-dependent (BOLD) response during a PL task (SRTT) following a switch to one of two atypical antipsychotics, risperidone and olanzapine, in patients previously on stable doses of typical antipsychotics. Risperidone and olanzapine were chosen over other atypical antipsychotics because they, despite both being classified as atypical antipsychotics, have relatively distinct pharmacological profiles (Arnt and Skarsfeldt 1998; Leysen et al. 1994; Miyamoto et al. 2008) and are also two of the most commonly prescribed antipsychotics to people with schizophrenia. We hypothesised that the substitution of atypical for typical antipsychotics would be associated with improved PL and restored activation of neural systems that subserve PL in healthy people. In addition, considering the different receptor profiles of atypical antipsychotics (Miyamoto et al. 2008), possible differences in PL and associated neural patterns between olanzapine and risperidone were explored.

## Method

### Participants and design

Thirty patients (aged 18–61 years) with a DSM-IV (Diagnostic and Statistical Manual of Mental Disorders, 4th edition) diagnosis (First et al. 1996) of schizophrenia participated. All included patients were required to be (a) on stable doses of typical antipsychotics for 6 or more weeks, (b) free from illicit drugs (confirmed with urine analysis), (c) strongly right-handed as determined using the Edinburgh Handedness Inventory (Oldfield 1971) and (d) able to provide written informed consent. Patients entering the study were examined clinically to ensure compliance with the inclusion and exclusion criteria.

The study was a single-centre, open-label study, with blinded ratings. Within 1 week of baseline assessment, out of 30 patients recruited into the study, 10 were randomised to remain on typical antipsychotics for the duration of this study, while 20 patients were randomly allocated to receive treatment with atypical antipsychotics olanzapine (*n* = 10) or risperidone (*n* = 10). The person responsible for randomisation was aware of the patients’ gender and age but unaware of the results of the baseline clinical and neuroimaging results. Starting doses for atypical antipsychotics were according to the “Summary of product characteristics” for each of the two drugs: 10 mg for olanzapine and 2 mg for risperidone. Optimal dose was achieved within 14 days, and patients were re-assessed after having been on the optimal dose for 6 weeks. The optimal dose range for risperidone was 4–8 mg and for olanzapine 10–20 mg. All participants underwent fMRI during the PL task on two occasions: at baseline and then again 7–8 weeks later. Severity of symptoms was rated with the Positive and Negative Syndrome Scale (PANSS) (Kay et al. 1987) on both occasions. Predicted IQ was assessed using the National Adult Reading test (NART) (Nelson and Willison 1991) at baseline for sample characterisation. The age of onset of illness and the type and doses of current antipsychotic treatment were also recorded. Data were unusable due to motion artefacts or image acquisition failures at baseline or follow-up for one patient of each of the risperidone and olanzapine groups and for two patients of the typical group. In addition, one patient of the risperidone group did not provide behavioural data (failed to press the button on most trials) during fMRI, and one patient of each of the olanzapine and risperidone groups did not attend their follow-up scan. The final sample thus comprised 23 patients: 8 on typical (at baseline, three on flupenthixol decanoate, four on zuclopenthixol decanoate and one on fluphenazine decanoate) and 15 on atypical antipsychotics [seven risperidone (at baseline, two on flupenthixol decanoate, three on zuclopenthixol decanoate, one on fluphenazine decanoate and one on trifluoperazine), eight olanzapine (at baseline, three on flupenthixol decanoate, two on zuclopenthixol decanoate, two on fluphenazine decanoate and one on sulpiride)] (Table 1).

**Table 1:** Descriptive statistics [mean, standard deviation (SD)] and analysis of variance results for demographics and clinical characteristics of patient groups and subgroups

	Typical (*n* = 8; 5 men)	Atypical (*n* = 15; 9 men)	ANOVA		Atypical subgroups		ANOVA	
			Group effect		Risperidone (*n* = 7; 5 men)	Olanzapine (*n* = 8; 4 men)	Group effect	
	Mean (SD)	Mean (SD)	*F*(1,21)	*p*	Mean (SD)	Mean (SD)	*F*(1,13)	*p*
Age (years)	46.00 (9.81)	38.60 (15.28)	1.52	0.22	35.57 (13.73)	41.25 (16.97)	0.50	0.49
Education (years)	11.25 (3.69)	12.73 (3.61)	0.87	0.36	12.29 (3.30)	13.13 (4.05)	0.19	0.67
Predicted (NART) IQ	96.37 (16.31)	103.33 (14.39)	1.11	0.30	100.57 (16.35)	105.75 (13.06)	0.47	0.51
Age at illness onset (years)	23.43 (5.62)	25.13 (10.66)	0.16	0.70	20.71 (3.90)	29.00 (13.33)	2.46	0.14
Duration of illness (years)	20.43 (9.88)	13.47 (12.75)	1.61	0.22	14.86 (14.75)	12.25 (11.63)	0.15	0.71
Antipsychotic dose in chlorpromazine equivalents (mg)	220.84 (146.04)	267.86^a^ (251.62)	0.23^b^	0.64	314.29 (337.53)	221.43^c^ (134.96)	0.46^c^	0.51

^a^Dose information missing for one patient

^b^df = 1,20

^c^df = 1,12

The study procedures were approved by the ethics committee of the Institute of Psychiatry and Maudsley Hospital, London. The participants provided written informed consent after the methods and procedure had been discussed with them.

### Experimental design and procedure

This study used the same PL task, i.e. a 5-min sequence learning task in a blocked periodic AB design, as used in a number of previous studies (Ettinger et al. 2013; Kumari et al. 2008; Kumari et al. 2002). Briefly stated, the task consisted of two 30-s alternating conditions: blocks of random trials (control condition) and blocks of pattern trials (experimental condition). In total, there were five 30-s random and five 30-s pattern conditions. Participants were presented with a white target stimulus (an asterisk) projected on to a black screen via a prismatic mirror fitted in the radiofrequency head coil as they lay in the scanner. The screen was divided into four equal quadrants by two intersecting white lines, and the target moved between these four locations on the screen.

For the pattern condition, the target movements were predictable for 75 % of cases, i.e. stimulus locations were determined following three specific rules: (1) a horizontal target movement was followed by a vertical target movement, (2) a vertical target movement was followed by a diagonal target movement and (3) a diagonal target movement was followed by a horizontal movement. The fourth movement of the target during the pattern condition was determined randomly, which then was followed by the abovementioned three specific rules.

Participants were required to follow each target movement with their right hand as fast as possible using a MRI compatible key pad with four keys, each key corresponding to one of the four quadrants. The movement of the target was initiated by the participants’ touching of the target key. RTs were recorded on-line. Participants were not told about the predetermined sequence during the pattern condition, and the beginning of random and pattern condition was not marked in any way. Prior to scanning, all participants underwent a practice session during which they practiced on five 30-s random and five 30-s pattern condition, both alternated with 30-s rest periods.

### Image acquisition

Echoplanar MR brain images were acquired using a 1.5 T GE Signa system (General Electric, Milwaukee, WI, USA) at the Maudsley Hospital, London. Daily quality assurance was carried out to ensure high signal-to-ghost ratio, high signal-to-noise ratio and excellent temporal stability using an automated quality control procedure (Simmons et al. 1999). A quadrature birdcage head coil was used for radio frequency transmission and reception. In each of 16 near-axial, non-contiguous planes parallel to the intercommissural (AC-PC) plane, 100 T2*-weighted MR images depicting blood oxygenation level-dependent (BOLD) contrast (Ogawa et al. 1990) were acquired over the 5 minute experiment with echo time (TE) = 40 ms, repetition time (TR) = 3 s, in-plane resolution = 3.1 mm, slice thickness = 7.0 mm, and interslice gap = 0.7 mm. Head movement was limited by foam padding within the head coil and a restraining band across the forehead. At the same session, a high-resolution 3-D inversion recovery prepared spoiled GRASS pulse sequence was used to acquire a T1-weighted volume in the axial plane (TR = 12.2 ms, TE = 5.3 ms, TI = 300 ms, flip angle = 20°, in-plane resolution = 0.94 mm, matrix dimensions 256 × 256), yielding 124 contiguous slices of 1.5 mm thickness.

### Data analysis

All demographic, clinical and behavioural measures were analysed using SPSS version 22.00 (SPSS Inc., Chicago, IL USA). Effect sizes, where reported, are partial eta squared (i.e., the proportion of variance associated with a factor). The α-level of significance (two-tailed) was set at *p* < 0.05 unless indicated otherwise.

### Demographic and clinical measures

Typical and atypical antipsychotic groups were compared on age, education and predicted IQ using one-way analysis of variance (ANOVA). The data on positive symptoms, negative symptoms, general psychopathology and total PANSS scores were analysed (separately) with a 2 (typical, atypical) × 2 (baseline, follow-up) repeated-measures ANOVA. Further, one-way ANOVAs were conducted to compare the two atypical antipsychotic subgroups (risperidone, olanazapine) on age, education and predicted IQ and 2 (risperidone, olanzapine) × 2 (baseline, follow-up) repeated-measures ANOVAs to examine changes in positive symptoms, negative symptoms, general psychopathology and total PANSS scores.

### Task performance

To examine group differences at baseline and follow-up, mean RTs to blocks of random and pattern trials at baseline and follow-up were subjected to a four-way group (2: typical, atypical) × occasion (2: baseline, follow-up) × trial type (2: random, pattern) × block (5: five 30-s blocks of random and pattern trials) ANOVA with group as a between-subjects factor, and occasion, trial type and block as within-subjects factors, followed by lower-order ANOVAs and post-hoc mean comparisons as appropriate. A further group (risperidone, olanzapine) × occasion × trial type × block ANOVA was carried out to explore possible differences between different atypical antipsychotics, followed by lower-order ANOVAs and post-hoc mean comparisons. Finally, we examined potential associations across patients, between age, illness duration and symptom levels and PL scores at baseline and follow-up using Pearson’s correlations.

### fMRI

Data were analysed using statistical parametric mapping software (SPM8; http://www.fil.ion.ucl.ac.uk/spm) running in MATLAB version 7.14 (The Math Works Inc).

### Image preprocessing

For each participant, the 100-volume functional time series were realigned to the first volume, corrected for motion artefacts, normalised to the Montreal Neurological Institute echo-planar imaging template, smoothed with an 8-mm full-width at half-maximum Gaussian filter and band-pass-filtered.

### Models and statistical inferences

fMRI data were analysed using a two-stage random-effects procedure (Friston et al. 1999). The first stage identified subject-specific task-related activations relevant to pattern trials (experimental condition) over the entire session as well as linear increases and decreases in activity over the five blocks of pattern trials. Random trials (control condition) served as the implicit baseline. The contrast images were obtained by using a boxcar design for each 30-s epoch convolved with the hemodynamic response function. Motion parameters obtained from the realignment pre-processing step were included as covariates at this stage. In the second stage of analysis, the resulting maps were used to establish task-related activations across subject-specific images using one-sample *t*-tests for each group (typical, atypical; risperidone, olanzapine) at baseline and follow-up. Significance was assessed with correction for multiple comparisons at the cluster level (*p* < 0.01 corrected). The pattern > random contrast revealed insufficient activations at the corrected level in all analyses (thus not reported hereafter). Further described analyses were conducted in contrasts with linear increases and decreases in activity over the five blocks of pattern trials. Significant changes from baseline to follow-up in atypical antipsychotic group were examined with group (typical, atypical) × occasion (baseline, follow-up) SPM ANOVA, followed by paired *t*-tests separately in the typical and atypical groups. We further explored the regions that were significantly active at follow-up following a switch to risperidone or olanzapine (separately) using paired *t*-tests. Significance was assessed using a correction for multiple comparisons at the cluster level (*p* < 0.05) with a height threshold of *p* = 0.01 for baseline-to-follow-up activation changes in the typical and atypical groups and *p* = 0.05 (to accommodate smaller *N*s and the exploratory nature of the analyses) in the risperidone and olanzapine subgroups.

## Results

### Demographic and clinical measures

Age, level of education, illness duration, age of onset and the dose of antipsychotic medication at baseline did not differ significantly between the typical and atypical antipsychotics group or between the two atypical antipsychotic subgroups (risperidone, olanzapine) (Table 1).

The change in symptom levels (from baseline to follow-up) was not significant for typical or atypical groups or for either of the two atypical antipsychotic subgroups (Table 2; all *p* values >0.05 for all group, occasion and group × occasion effects).

**Table 2:** Descriptive statistics [mean, standard deviation (SD)] and analysis of variance results for symptom measures and task performance at baseline and follow-up

	Typical (*n* = 8)		Atypical (*n* = 15)		ANOVA						Risperidone (*n* =7)		Olanzapine (*n* = 8)		ANOVA					
	Baseline	Follow-up	Baseline	Follow-up	Group	Occasion	Group × occasion				Baseline	Follow-up	Baseline	Follow-up	Group		Occasion		Group × occasion	
PANSS symptoms^a^	Mean (SD)	Mean (SD)	Mean (SD)	Mean (SD)	*F*(1,21),	*p*	*F*(1,21)	*p*	*F*(1,21)	*p*	Mean (SD)	Mean (SD)	Mean (SD)	Mean (SD)	*F*(1,13)	*p*	*F*(1,13)	*p*	*F*(1,13)	*p*
Positive symptoms	14.75 (6.02)	13.62 (6.43)	19.07 (6.66)	15.86 (5.05)	1.93	0.18	3.37	0.08	0.77	0.39	18.29 (8.36)	13.86 (4.49)	19.75 (5.26)	17.62 (5.12)	1.05	0.33	3.87	0.07	0.48	0.5
Negative symptoms	17.37 (4.17)	15.25 (5.55)	19.47 (6.44)	18.80 (6.74)	1.34	0.26	1.68	0.21	0.46	0.5	18.57 (6.24)	19.14 (7.69)	20.25 (6.94)	18.50 (6.32)	0.02	0.88	0.2	0.66	0.79	0.39
General psychopathology	36.37 (9.16)	33.87 (9.55)	40.40 (10.04)	35.67 (9.64)	0.61	0.44	3.16	0.09	0.3	0.59	39.57 (9.96)	34.29 (10.37)	41.12 (10.74)	36.87 (9.51)	0.22	0.64	2.74	0.12	0.03	0.86
Total symptoms	68.50 (16.13)	62.75 (18.52)	78.93 (20.23)	70.33 (19.51)	1.46	0.24	3.59	0.07	0.14	0.71	76.43 (22.01)	67.29 (21.65)	81.12 (19.79)	73.00 (18.50)	0.01	0.92	2.7	0.12	0.01	0.92

^a^Positive and Negative Syndrome Scale (PANSS; Kay et al. 1987)

### Task performance

The four-way repeated-measures ANOVA (group × occasion × trial type × block) revealed a significant main effect of block [*F*(4,84) = 4.76, *p* =0.008, ηρ^2^ = 0.18] and a significant interaction between occasion × trial type [*F*(1,21) = 6.84, *p* =0.02, ηρ^2^ = 0.25]. The group × trial type × occasion interaction failed to attain formal statistical significance (with small *N*/group), but it had a moderate effect size [*F*(1,21) = 2.51, *p* = 0.13, ηρ^2^ = 0.11], and the possible effects of typical and atypical antipsychotics on PL were explored to inform our hypotheses as noted below.

The three-way repeated-measures ANOVA (group × trial type × block) on baseline data revealed non-significant effects involving group, trial type × block [trial type, *F*(1,21) = 0.26, *p* = 0.62, ηρ^2^ = 0.01; block × trial type, *F*(1,21) = 0.04, *p* =0.85, ηρ^2^ = 0.002; trial type × group, *F*(1,21) = 0.04, *p* = 0.85, ηρ^2^ = 0.002], confirming no difference between RTs to random and pattern trials during any of the blocks in typical or atypical antipsychotics groups at baseline (when all patients were on typical antipsychotics). There was only a main effect of block [*F*(4,84) = 3.60, *p* = 0.03, ηρ^2^ = 0.15] with a linear effect [*F*(1,21) = 5.52, *p* = 0.029, ηρ^2^ = 0.21], indicating a reduction in RTs from block 1 to block 5 (of both random and pattern trials) across all patients at baseline (Table 3).

**Table 3:** Task performance [mean, standard error of the mean (SEM)] of patient groups and sub-groups at baseline and follow-up

		Typical (*n* = 8)		Atypical (*n* = 15)		Risperidone (*n* =7)		Olanzapine (*n* = 8)	
Task performance		Baseline	Follow-up	Baseline	Follow-up	Baseline	Follow-up	Baseline	Follow-up
Reaction time (ms)		Mean (SEM)	Mean (SEM)	Mean (SEM)	Mean (SEM)	Mean (SEM)	Mean (SEM)	Mean (SEM)	Mean (SEM)
Block 1	Random trials	0.348 (0.075)	0.308 (0.063)	0.296 (0.055)	0.338 (0.0465)	0.230 (0.025)	0.322 (0.082)	0.355 (0.072)	0.352 (0.059)
	Pattern trials	0.426 (0.089)	0.314 (0.056)	0.322 (0.065)	0.301 (0.041)	0.223 (0.017)	0.278 (0.052)	0.408 (0.099)	0.321 (0.055)
Block 2	Random trials	0.433 (0.085)	0.302 (0.055)	0.353 (0.062)	0.335 (0.040)	0.232 (0.026)	0.305 (0.058)	0.459 (0.094)	0.361 (0.055)
	Pattern trials	0.447 (0.075)	0.303 (0.059)	0.308(0.055)	0.318(0.043)	0.218 (0.020)	0.296 (0.075)	0.387 (0.066)	0.337 (0.048)
Block 3	Random trials	0.430 (0.077)	0.292 (0.062)	0.319 (0.056)	0.343 (0.046)	0.218 (0.033)	0.339 (0.010)	0.407 (0.070)	0.347 (0.035)
	Pattern trials	0.469 (0.091)	0.286 (0.052)	0.330 (0.067)	0.301 (0.038)	0.206 (0.027)	0.290 (0.070)	0.438 (0.082)	0.310 (0.036)
Block 4	Random trials	0.466 (0.081)	0.295 (0.052)	0.302 (0.059)	0.317 (0.038)	0.192 (0.032)	0.283 (0.059)	0.399 (0.061)	0.347 (0.037)
	Pattern trials	0.448 (0.083)	0.296 (0.051)	0.314 (0.061)	0.288 (0.037)	0.197 (0.024)	0.256 (0.054)	0.416 (0.068)	0.317 (0.039)
Block 5	Random trials	0.393 (0.068)	0.267 (0.048)	0.294 (0.049)	0.316 (0.035)	0.194 (0.036)	0.300 (0.060)	0.381 (0.054)	0.330 (0.032)
	Pattern trials	0.395 (0.076)	0.270 (0.047)	0.303 (0.056)	0.282 (0.0346)	0.197 (0.029)	0.254 (0.036)	0.395 (0.058)	0.307 (0.034)

**Fig. 1:**
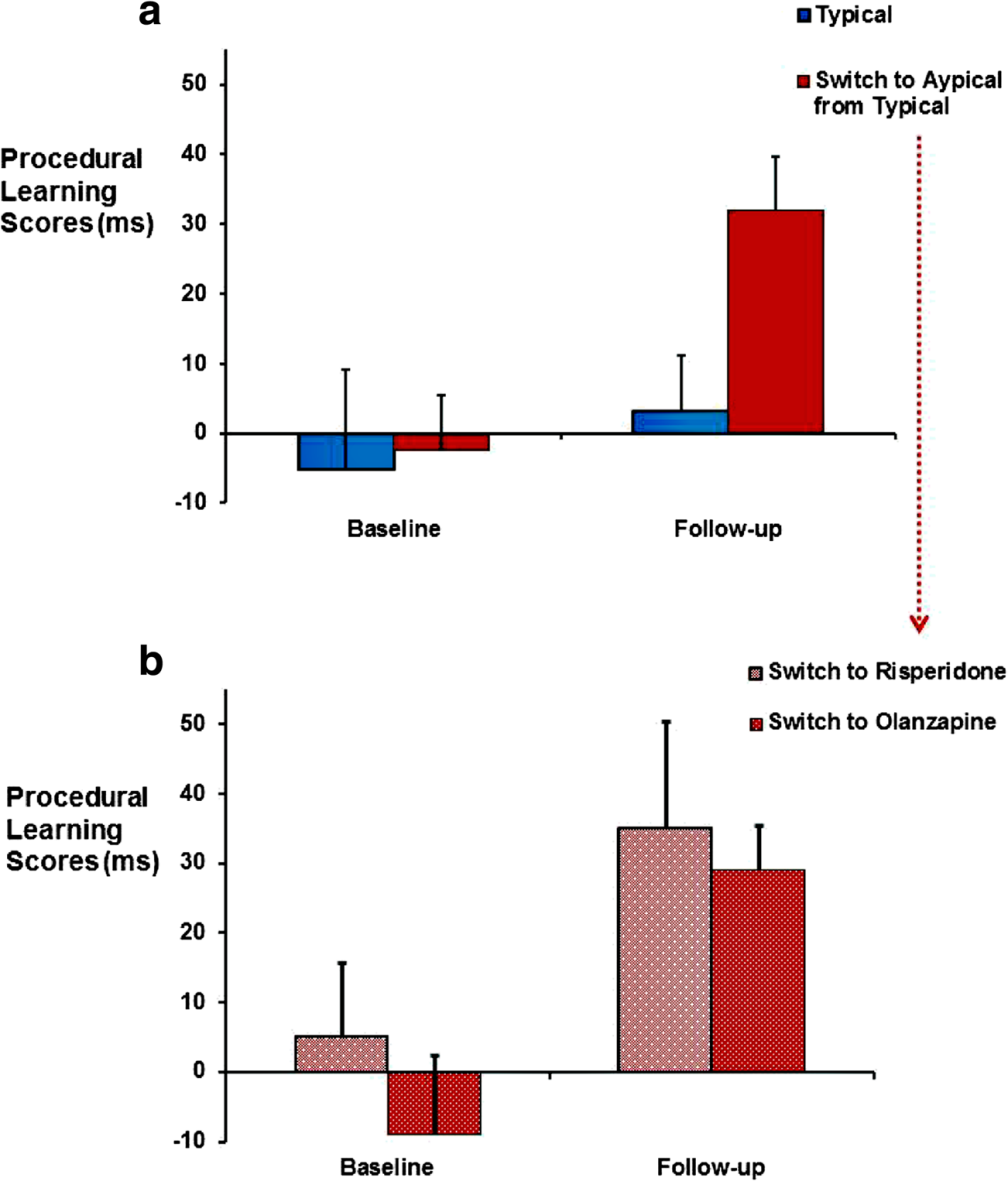
**a** Mean (+1 standard error of the mean) procedural learning scores (mean reaction times over five blocks of pattern trials *minus* mean reaction times over five blocks of random trials) at baseline and follow-up in the group that remained on typical antipsychotics and the group that was switched to atypical antipsychotics (risperidone or olanzapine). **b** Mean (+1 standard error of the mean) procedural learning scores at baseline and follow-up in patients switched from typical antipsychotics to risperidone and olanzapine

The three-way repeated-measures ANOVA (group × trial type × block) on follow-up data showed a significant main effect of trial type [*F*(1,21) =8.65, *p* = 0.008, ηρ^2^ = 0.29] and importantly a significant trial type × group (typical, atypical) interaction [*F*(1,21) = 5.77, *p* = 0.03, ηρ^2^ = 0.22]. Separate analysis of typical and atypical group confirmed no effect of trial type in the typical group [*F*(1,7) = 0.17, *p* = 0.70, ηρ^2^ = 0.02] but a strong main effect of trial type in the atypical group [*F*(1,14) = 17.53, *p* =0.001, ηρ^2^ = 0.56], showing faster RTs over pattern trials relative to random trials (Table 3; Fig. 1). A significant main effect of block [*F*(4,84) = 2.95, *p* = 0.05, ηρ^2^ = 0.12] with a linear decrease in RT over the five blocks of trials [*F*(1,21) = 6.09, *p* = 0.02, ηρ^2^ = 0.22] was also present, but there was no significant interaction involving the block factor (*p* >0.05).

The three-way repeated-measures ANOVA (occasion × trial type × block) for the typical antipsychotic group yielded no significant effects of trial type [*F*(1,7) = 0.01, *p* = 0.91 , ηρ^2^ = 0.002], occasion [*F*(1,7) = 1.14, *p* = 0.32, ηρ^2^ = 0.14] or occasion × trial type [*F*(1,7) = 0.29, *p* = 0.61, ηρ^2^ = 0.04], indicating that patients who remained on typical antipsychotics did not show RT reduction to pattern trials relative to random trials at baseline or follow-up. The three-way repeated-measures ANOVA (occasion × trial type × block) for the atypical group showed a significant main effect of trial type [*F*(1,14) = 5.30, *p* = 0.04, ηρ^2^ = 0.27] and a significant interaction effect of occasion × trial type [*F*(1,14) = 6.08, *p* = 0.001, ηρ^2^ = 0.53]. Follow-up of this (occasion × trial type) interaction showed no effect of trial type or block at baseline (*p* values >0.20) but a highly significant main effect of trial type [*F*(1,14) = 17.54, *p* = 0.001, ηρ^2^ = 0.56], showing much faster RTs during pattern trials than random trials over the entire experiment (random trials: mean = 0.33 s, SEM = 0.39; pattern trials: mean = 0.298 s, SEM = 0.034) at follow-up in this group (Table 3).

Exploration of the atypical subgroups (risperidone, olanzapine) revealed comparable reduction in RTs to pattern trials relative to random trials at follow-up as demonstrated by a significant occasion × trial type interaction [*F*(1,13) = 14.86, p = 0.002, ηρ^2^ = 0.53], but no significant group [*F*(1,13) = 3.06, *p* = 0.10, ηρ^2^ = 0.19], group × trial type [*F*(1,13) = 0.58, *p* = 0.46, ηρ^2^ = 0.04] or group × occasion × trial type effect [*F*(1,13) = 0.20, *p* =0.66, ηρ^2^ = 0.01].

Age and illness duration did not correlate with PL scores at baseline (age: *r* = −0.12, *p* = 0.58; illness duration: *r* = 0.10, *p* = 0.66) or follow-up (age: *r* = −0.03, *p* = 0.88; illness duration: r = 0.07, *p* = 0.76). PANSS symptom ratings and PL scores were also not significantly correlated at baseline (positive symptoms: *r* = −0.19, *p* = 0.30; negative symptoms: *r* = −0.08, *p* = 0.69; general psychopathology: *r* = −0.16, *p* = 0.45; total symptoms: *r* = −0.04, *p* = 0.85) or follow-up (positive symptoms: *r* = −0.09, *p* = 0.68; negative symptoms: *r* = −0.14, *p* = 0.52; general psychopathology: *r* = −0.19, *p* = 0.39; total symptoms: *r* = −0.17, *p* = 0.45).

### fMRI

#### Generic task-related activations

##### Baseline

In line with the behavioural results, there were no significant task-related activations in typical or atypical groups when patients were all on typical antipsychotics.

##### Follow-up

Patients who remained on typical antipsychotics did not show any significant activation changes also at follow-up. In those who were switched to atypical antipsychotics, activity in the superior-middle frontal and anterior cingulate increased linearly over the five blocks of pattern trials (Table 4). When explored separately in the risperidone and olanzapine subgroups, the risperidone subgroup showed a significant activity increase over the five blocks in the lentiform nucleus extending to the insula (Table 4). The olanzapine subgroup showed a significant activity increase over the five blocks in the caudate extending to the anterior cingulate and precentral gyrus (Table 4). No area showed activation decreases over the five blocks of pattern trials.

**Table 4:** Brain areas showing significant activation increases (over block 1 to block 5 of pattern, relative to random, trials) in the atypical antipsychotics group at follow-up

Brain region	BA	Side	MNI coordinates			Voxel *T* value	Cluster size (voxel *n*)	Cluster-corrected *p*
			*x*	*y*	*z*			
Atypical antipsychotics (across risperidone and olanzapine) (height threshold *p* = 0.01)								
Superior frontal gyrus	10	R	36	58	2	4.91	1259	0.016
	10	R	24	60	6	3.41		
	10	R	24	52	16	3.10		
Middle frontal gyrus	10/46	R	34	56	16	3.68		
Anterior cingulate/medial frontal gyrus	32/10	R	2	54	14	3.46		
Separately for risperidone and olanzapine (height threshold *p* = 0.05)								
Risperidone								
Lentiforn nucleus	n/a	R	24	−20	−4	7.75	4496	0.001
Insula	n/a	R	34	24	8	7.23		
Superior frontal gyrus	10	R	32	48	−2	3.48		
Olanzapine								
Caudate	n/a	R	6	2	10	4.97	5879	0.01
Anterior cingulate	24/32	R	12	32	20	4.66		
Precentral gyrus	6	R	36	−2	28	4.49		

*BA* Brodmann area, *MNI* Montreal Neurological Institute, *R* right, *L* left

#### Activation changes from baseline to follow-up

**Fig. 2:**
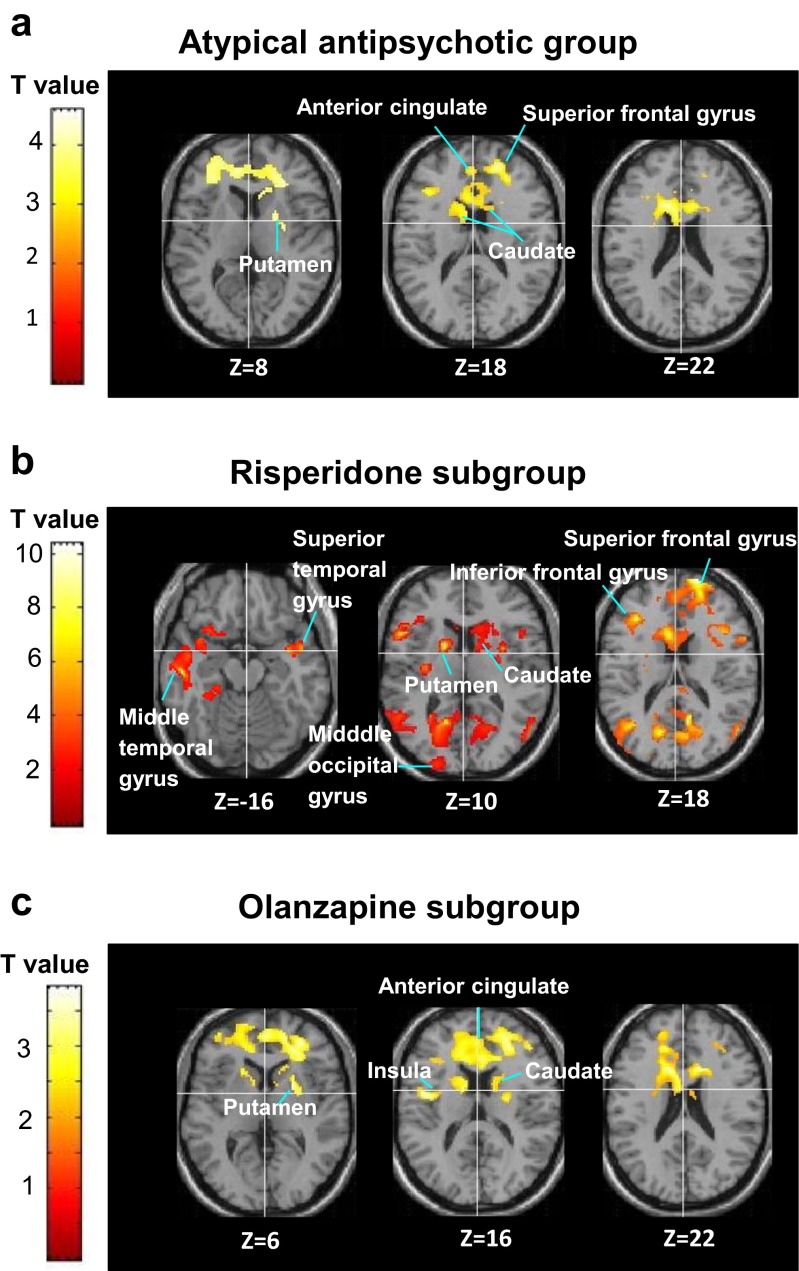
**a** Areas of increased brain activity following atypical antipsychotic treatment (height threshold *p* < 0.01) in axial views with associated MNI *z* coordinates. Left hemisphere is shown on the *left*. **b**, **c** Areas of increased brain activity separately for atypical antipsychotic subgroups—risperidone and olanzapine (height threshold *p* <0.05)

There were no activation increases or decreases (at the corrected or uncorrected cluster level) from baseline to follow-up in those who remained on typical antipsychotics. Following a switch to atypical antipsychotics, increased activation, relative to baseline, was seen in one cluster in the bilateral superior frontal gyrus extending to the inferior frontal gyrus and in another cluster in the left anterior cingulate, extending to the right putamen and bilateral caudate (Table 5; Fig. 2a); no area showed a significant decrease in activity from baseline to follow-up.

**Table 5:** Brain areas showing significant activation increases (over block 1 to block 5 of pattern, relative to random, trials) from baseline to follow-up after a switch to atypical antipsychotics (height threshold *p* = 0.01)

Brain region	BA	Side	MNI coordinates			Voxel *T* value	Cluster size (voxel *n*)	Cluster *p*
Following a switch to atypical antipsychotics risperidone or olanzapine (height threshold *p* = 0.01)								
Superior frontal gyrus	10	R	20	50	18	4.26	1115	0.007
	10	L	−22	50	6	3.39		
Inferior frontal gyrus	47	R	30	34	4	3.56		
	10	L	−40	52	6	3.27		
Anterior cingulate	32	L	−14	18	22	4.6	851	0.029
Putamen		R	22	4	8	3.67		
Caudate		R	14	26	8	3.21		
		L	−16	6	14	3.07		
Separately for risperidone and olanzapine (height threshold *p* = 0.05)								
Following switch to risperidone								
Posterior cingulate	30	L	−16	−60	4	11.31	6037	<0.001
	30	R	10	−58	18	6.14		
Middle occipital gyrus	19	L	−22	−68	4	7.72		
Paracentral lobule	5	R	18	−40	54	7.14		
	5	L	−6	−44	48	6.80		
Precuneus	19	L	−32	−78	38	6.69		
Putamen	n/a	L	−16	6	10	10.35	2029	*0.002*
	n/a	R	32	4	10	5.83		
Caudate	n/a	L	−12	12	2	5.68		
Insula	n/a	L	−34	−12	8	5.37		
Cingulate gyrus	24	R	4	−10	26	5.17		
	24	L	−2	−6	28	3.87		
Anterior cingulate	32	L	−2	12	26	4.24		
Superior temporal gyrus	22	L	−48	−16	−8	9.89	1097	*0.018*
Inferior frontal gyrus	47	L	−46	24	0	9.73		
Middle frontal gyrus	9	L	−30	30	22	6.75		
Middle temporal gyrus	21	R	54	−18	−16	9.91	1033	*0.021*
Parahippocampal gyrus	36	R	46	−32	−10	5.85		
Superior frontal gyrus	10	R	18	66	18	6.12	811	*0.037*
Following switch to olanzapine								
Anterior cingulate	32	L	−14	18	22	3.84	7429	< 0.001
	32	R	12	18	22	3.55		
Precentral gyrus	6	L	−36	−2	28	3.55		
Cingulate gyrus	32	R	18	4	46	3.48		
Superior frontal gyrus	6	R	2	6	58	3.45		
Middle frontal gyrus	9	R	38	28	42	3.43		
	8	L	−26	30	52	3.43		
Insula	13	L	−46	−2	16	3.41		
Caudate		L	−6	4	20	3.37		
		R	4	6	8	3.37		
Putamen		L	−18	18	2	3.3		

Cluster *p* in italics: uncorrected. All others: cluster *p* corrected for multiple comparisons across the entire brain

*BA* Brodmann area, *MNI* Montreal Neurological Institute, *R* right, *L* left

During exploration of the two atypical groups separately, the risperidone group showed activation increases in a large cluster in the posterior cingulate, extending to the left middle occipital gyrus, bilateral paracentral lobule and left precuneus (Table 5). Additional activation increases, at the uncorrected cluster level, were found in the putamen (bilaterally) extending to the left caudate, bilateral insula and bilateral cingulate gyrus; the left superior temporal gyrus extending to the left inferior-middle frontal gyrus; the right middle temporal gyrus extending to the right parahippocampal gyrus; and the right superior frontal gyrus (Table 5; Fig. 2b). In the olanzapine group, there was increased activity in the bilateral anterior cingulate (bilateral), extending to the left precentral gyrus, right superior and bilateral middle frontal gyrus, left insula, bilateral caudate and left putamen (Table 5; Fig. 2c). No area showed a significant decrease in activity from baseline to follow-up in the risperidone or olanzapine subgroups.

## Discussion

To our knowledge, this is the first longitudinal fMRI study examining the neural effects associated with switching schizophrenia patients from typical to atypical antipsychotics using a fMRI-compatible PL task. We also explored the possible differentiation between risperidone and olanzapine.

### Behavioural findings

At baseline, patients (all on typical antipsychotics) did not show PL (i.e. no significant difference between RTs to random and pattern trials); there was also no difference in PL of patients who remained on typical antipsychotics and those who were later switched to receive risperidone or olanzapine. At follow-up, there was a difference between the typical and atypical groups that was in accordance with our a priori hypotheses, with significantly faster RTs to pattern, relative to random, trials in those on atypical (but not typical) antipsychotics.

An aspect of the experiment deserving comment is that although significantly faster RTs to pattern, relative to random, trials reflect PL in the atypical group at follow-up, this difference was present even during the very first block of trials, suggesting that patients on atypical antipsychotics had learnt the sequence during the first block itself, perhaps due to the practice session conducted just prior to scanning. This was also observed earlier in a healthy group with the same task and administration procedures (Kumari et al. 2002), but it may also mean that the neural findings of this study relate to the recall, rather than acquisition, of implicit knowledge about the sequences.

The pattern of results obtained in the typical and atypical antipsychotic groups at baseline and follow-up is consistent with the finding of previous studies using SRTT (Green et al. 1997; Kumari et al. 2008; Kumari et al. 2002) and also of those using other PL tasks (Paquet et al. 2004; Purdon et al. 2003; Scherer et al. 2004). Specifically, studies have reported normal PL on a visual tracking task in patients on olanzapine, but not in those on haloperidol (Paquet et al. 2004); normal PL on a mirror drawing task was shown in those on clozapine or risperidone, but not in those on haloperidol (Scherer et al. 2004), and preserved PL, defined as the improvement observed between two blocks of five trials of the Tower of Toronto, following 6 months of treatment with olanzapine but a decline following the 6-month use of risperidone or typical antipsychotics in medication-naïve patients (Purdon et al. 2003). Atypical antipsychotic treatment-related PL changes apparent at follow-up may at least in part represent a functional consequence of the robust antagonism of D_2_ receptors in the striatum by typical antipsychotics at baseline (Bedard et al. 1996; Kumari et al. 2002) and less so at follow-up. The inconsistency of results in previous studies regarding risperidone use may be explained by the varying doses of risperidone and sample characteristics in previous studies. Risperidone, when given at doses of more than 8 mg/daily, produces an extrapyramidal symptoms profile (Lemmens et al. 1999) and D_2_ blockade broadly similar to that of typical antipsychotics (Marder and Meibach 1994). The effects of risperidone at a given dose are also known to be affected by illness chronicity. Maximum antipsychotic activity occurs at 4–6 mg/day for chronic schizophrenia patients, whereas first-episode patients respond to lower (2–5 mg/day) doses, and in chronic patients, daily doses of up to 8 mg/day are not associated with a greater risk of extrapyramidal symptoms than placebo (Foster and Goa 1998). The patients included in our study were chronically ill, and no patient was on more than 8 mg daily dose of risperidone (six of eight patients on 4–6 mg/day). This may explain improved PL in risperidone-treated patients at follow-up in our study.

Previously, it has been suggested that PL deficits may be linked to a symptomatic state (Exner et al. 2006). However, absent PL in the typical antipsychotic group does not seem to be related to clinical manifestation of symptoms or attentional difficulties since their RTs improved over successive blocks. It is likely that task familiarity or practice contributed to RT learning in typical antipsychotic patients since faster RT over blocks 1 to 5 occurred only at follow-up. Patients on typical antipsychotics have been suggested to rely on RT learning, whilst healthy controls tend to switch from RT learning to pattern learning towards later trials (Green et al. 1997). Improved PL despite non-significant symptom changes at follow-up in patients who were switched to atypical antipsychotics also suggests lack of a direct relationship between PL and symptom levels in stable schizophrenia patients.

### Neural changes following a switch to atypical antipsychotics

Typical antipsychotics use was associated with deficient task-related activation at baseline and follow-up. This complements our behavioural results of absent PL and replicates a previous fMRI study involving male patients only (Kumari et al. 2002). As anticipated, 6-week atypical antipsychotic treatment was associated with increased activation (over the five successive blocks of pattern trials) in the superior frontal gyrus extending to the inferior frontal gyrus and anterior cingulate extending to the caudate and putamen. Importantly, the areas showing activation increases following atypical antipsychotic treatment closely resemble those shown by healthy controls (Kumari et al. 2008; Purdon et al. 2011; Reiss et al. 2006; Zedkova et al. 2006). It is thus possible that the lack of PL and the lack of task-related activations at baseline were due, at least in part, to the potent D_2_ blocking mechanism of typical antipsychotics as suggested previously (Bedard et al. 1996; Kumari et al. 1997; Kumari et al. 2002). Interestingly, increased activation in the superior–inferior frontal gyrus, anterior cingulate and striatum was present as a linear increase over five successive 30-s blocks of sequenced trials. This observation, taken together with an earlier finding showing that clozapine-treated patients show progressive PL over successive trials whereas those treated with haloperidol show a high degree of fluctuation over trials (Bedard et al. 1996), indicates that a linear contrast may be more useful in characterising the incapacity to automate pattern learning in typically treated patients or a better capacity to reach optimal performance over a 5-min period in atypically treated patients.

#### Exploration of neural changes following a switch to risperidone and olanzapine

When baseline-to-follow-up neural changes were explored separately in the two atypical subgroups, partially overlapping and idiosyncratic activation changes emerged. Despite equivalent behavioural performance, the risperidone group activated regions in the posterior cingulate extending to middle occipital gyrus, paracentral lobule and precuneus followed by non-significant activation changes in the putamen, caudate, insula, cingulate gyrus, temporal regions and superior frontal gyrus. Increased activity in the posterior cingulate and middle temporal gyrus [in ziprasidone-treated individuals (Kumari et al. 2008)] and the precuneus [in healthy controls (Kumari et al. 2002)] has been shown to have a direct positive association with PL magnitude. Temporal regions have been suggested to play a compensatory role to overcome fronto-striatal deficits in schizophrenia and thereby achieve comparable PL as healthy controls (Zedkova et al. 2006). The risperidone-treated group failed to activate the striatal regions at the corrected level (possibly due to higher D_2_ blocking properties of risperidone compared to olanzapine), further supporting that the compensatory action of alternate regions contributed to the observation of improved PL at follow-up in this group.

Following a switch to olanzapine, significant task-related activation changes were detected in the anterior cingulate extending to the precentral gyrus, superior frontal gyrus, middle frontal gyrus, insula and striatum. Activation of this neural network is consistent with previous studies following successful PL in healthy controls (Kumari et al. 2008; Kumari et al. 2002; Purdon et al. 2011; Zedkova et al. 2006). This focused activation of PL-related regions possibly contributed to the PL-normalising effects of olanzapine.

## Limitations

First, this study is limited by unequal groups and small sample size and subsequent lack of robust statistical power. However, switching the same patients from typical to atypical antipsychotics reduced inter-individual differences. A second limitation may be the inclusion of patients on a range of oral and depot typical antipsychotics, introducing a potential source of heterogeneity. Previous studies have suggested that various typical antipsychotics may have different neural and cognitive effects depending on their potency (Abbott et al. 2011; Keefe et al. 2007a). Future studies could include low-potency typical antipsychotics when comparing with atypical antipsychotics. Third, the duration of exposure to atypical antipsychotics was short. Since PL has been shown to decline after 6 months of treatment with risperidone but not with olanzapine (Purdon et al. 2003), longer-term follow-up is needed for medication to be stabilised and to help elucidate how medication-induced changes in neural function evolve over time. Fourth, this study, as also mentioned earlier, may have identified neural effects associated with recall, and not acquisition, of implicit knowledge about the sequences. Further research with more sophisticated analytic strategies and longer exposures (e.g. with inclusion of the practice session and repeated presentation of the task) is required to explore this possibility. Practice session data in all baseline and follow-up sessions were not systematically recorded; thus, the exact effects of the practice session on baseline or follow-up task performance could not be examined in the present study. Further research could also use different experimental designs to separate the RT (fine motor) and sequence learning (cognitive) components, the latter of which is more likely to involve higher cortical brain regions, and include an additional group of treatment-naïve schizophrenia patients to clarify the effects of typical antipsychotics on PL and related brain activations. Fifth, given the dose-dependent actions of atypical antipsychotics on dopamine D_2_ receptors and in addition on many other receptors (Arnt and Skarsfeldt 1998; Foster and Goa 1998) and the absence of receptor imaging data in this study, our suggestion of reduced antagonism of D_2_ receptors at follow-up (relative to baseline) in those switched to risperidone or olanzapine as the potential mechanism for improved PL seen in this study remains speculative and requires further study. Finally, although not directly relevant to the aims of this study, it would have been informative to study a matched healthy control group over two occasions and establish healthy activation patterns in a test–retest design.

## Conclusions

Our findings of absent PL and deficient task-related activation at baseline and follow-up in the typical antipsychotic group and normalisation of PL and restoration of PL-related regions following a switch to atypical antipsychotics suggest that PL deficits may be secondary to treatment with typical antipsychotics via potent D_2_ blocking mechanism. Substituting risperidone and olanzapine may have different effects on brain function, and this in turn may relate to the differences in their receptor binding long-term clinical profiles. The present findings suggest that SRTT combined with fMRI may provide a useful biomarker for exploring the effects of medication on PL and emphasise the importance of considering medication status and antipsychotic type in neuroimaging studies.
